# Accelerating Replica Exchange Molecular Dynamics: A Comparison of Hydrogen Mass Repartitioning and Light Water Models

**DOI:** 10.1021/acs.jctc.5c01929

**Published:** 2026-01-09

**Authors:** Steven R. Bowers, William Jeffries, Christopher Lockhart, Dmitri K. Klimov

**Affiliations:** School of Systems Biology, 3298 George Mason University, Manassas, Virginia 20110, United States

## Abstract

Accelerating conformational sampling through changes in molecular mass is an attractive option in biomolecular modeling. Here, we examine the utility and compare the efficiency of hydrogen mass repartitioning (HMR) and light water (LW) models in the context of replica exchange (RE) simulations of an alanine dipeptide. To maintain integrator stability, we introduced scaling of integration steps with RE temperatures and determined their maximum values, assuring the stability of RE simulations. HMR2 and HMR3 models featuring doubled and tripled hydrogen masses and, to a lesser extent, the LW model reproduce the energetic and conformational properties of alanine dipeptide in water compared to the HMR1 reference. This conclusion is based on comparing kinetic and potential energies, free energy landscapes of the peptide, as well as its structural properties, including hydrogen bonding, water counts in the peptide first solvation shell, and RMSD distributions. Thereby, our results demonstrate that both HMR and LW models can be integrated into RE simulations. We then compared HMR and LW models with respect to the computational efforts required to equilibrate alanine dipeptide. HMR2 and HMR3 are up to 4-fold more computationally efficient than the HMR1 reference, whereas LW lags behind being less than a factor of 2 more efficient. As a result, LW efficiency is 2-fold lower than that of HMR3. This outcome means that increasing the integration step provides faster sampling than boosting water diffusion. Even if the computation of long-range interactions is adjusted with the length of the integration step and the step in LW simulations is further increased, the model remains less efficient than HMR3. We considered a hybrid variant of LW, hLW, featuring heavier water and mass repartitioning applied to all hydrogens, affording longer integration steps than LW does. hLW improves computational efficiency and provides more accurate reproduction of energetic and conformational properties of alanine dipeptide than LW. We concluded that HMR3 and hLW models demonstrate good performance in replica exchange simulation, but the former is preferable due to broader applicability and simplicity. hLW remains an excellent alternative to HMR3, but its scope is limited to “water-rich” systems. More generally, our findings suggest that among the two approaches, HMR or decreasing water mass, the former is more effective. Since LW simulations are not currently supported out-of-the-box by the NAMD molecular dynamics program, we implemented a patch enabling LW functionality.

## Graphical abstract

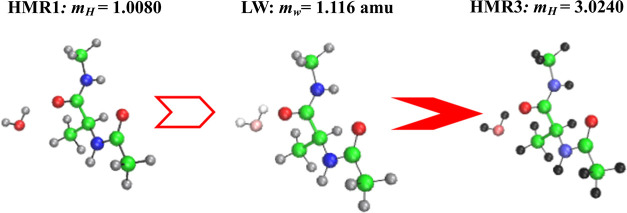

## 1 Introduction

Molecular dynamics (MD) simulations use all-atom models, atomic-level interaction forces, and Newton equations of motion to represent the behavior of biomolecular systems. Its distinctive feature lies in the bottom-up approach in which microscopic atomistic properties are linked to macroscopic quantities. However, typical biomolecular systems exhibit rugged free energy landscapes with high free energy barriers between multiple free energy minima of comparable depth. Consequently, to achieve an accurate thermodynamic description, MD must produce unbiased sampling of all relevant states and cross the barriers between them. Standard MD performed at a constant physiological temperature in all likelihood would fail to deliver ergodic sampling because it is hampered by exponentially long wait times for crossing free energy barriers. As a result, standard MD is often inadequate for probing complex biomolecular processes, including protein folding, protein–protein interactions, or protein binding to lipid bilayers.^1,2^

A partial solution to the problem outlined above is provided by enhanced sampling algorithms such as replica exchange molecular dynamics (REMD).^3–6^ REMD considers *R* distinct conditions, e.g., temperatures, and *R* system’s replicas assigning each of them to a specific condition. The key step in REMD implementation is periodic probabilistic swaps of replicas over adjacent conditions, generating a random walk of replicas across conditions. Since the collection of *R* replicas represents an extended canonical system, sufficiently long REMD simulations rigorously converge to equilibrium Boltzmann distributions. Over the years, REMD and its variants such as replica exchange with solute or hybrid tempering have been applied to study the thermodynamics of various biomolecular systems.^7–17^

However, even sophisticated implementations of replica exchange simulations do not guarantee converged biomolecular simulations.^18^ Consequently, additional methods must be applied to boost conformational sampling. One straightforward approach is to increase the length of the integration step *δt*. The typically used *δt* of 1 fs is limited by the high-frequency motions of hydrogen atoms. The integration step can be increased to 2 fs with the application of the restraints, which fix the lengths of hydrogen-related covalent bonds. In particular, NAMD and AMBER utilize iterative SHAKE algorithm to restrain these bonds in nonwater molecules, while the analytical SETTLE algorithm makes water molecules rigid. However, further increase in *δt* becomes problematic due to SHAKE instability.^19^ A possible solution to this limitation is the increase in the mass of hydrogen atoms to dampen their high-frequency motions. Although the first reference to this approach goes back to Jacucci and Raman in the 70 s,^20^ its thorough investigation has started significantly later.^19,21^ The premise of changing the hydrogen mass is based on the separation of kinetic and potential energy terms in the configurational integral. As a result, the thermodynamic value of a quantity *A*(*x*), which is the function of atomic coordinates *x*, is independent of atomic masses. Thus, as long as MD simulations use classical force fields without magnetic terms, this outcome holds.

It must be pointed out that a simple increase in hydrogen mass leading to heavier water molecules decreases the solvent diffusion coefficient and lengthens simulation time scales.^19,22^ Thereby, a more efficient solution referred to as hydrogen mass repartitioning (HMR) would increase the hydrogen mass but reduce the masses of heavy atoms, keeping the total molecule mass constant. Berendsen and co-workers have shown that HMR applied to all hydrogen atoms allows one to increase the integration step to 4 fs in the simulations of solvated protein provided that hydrogen-related covalent bonds are restrained.^19^ A more recent study of Roitberg and co-workers confirmed this finding by applying HMR either to all hydrogens or exclusively to water.^21^ Specifically, the authors determined that for the TIP3P water model and ff12SB force field, the integration step of *δt* = 4 fs affords stable MD simulations on a nanosecond scale. Critically, HMR does not skew structural, energetic, or kinetic properties of solvated peptide or protein. This conclusion was based on the comprehensive analysis of RMSD distributions, Ramachandran plots, potential mean force profiles, and rates of transitions between rotameric states. HMR can also be used to accelerate the MD simulations of lipid bilayers with embedded proteins without appreciable changes in structural properties.^23^ Previous studies have also identified subtle consequences of HMR. It has been found that the HMR, which triples hydrogen mass, increases the water moment of inertia causing about 10% decrease in water diffusion coefficient.^19^ Consequently, the gain in conformational sampling offered by HMR is 15% lower than that expected. More recently, Jung et al. showed that HMR can be combined with multistepping algorithms giving consistent results between the traditional simulations with *δt* = 2 fs and those utilizing the time steps of 3.5 fs for fast motions and 7.0 fs for slow motions.^24^ However, some studies challenged the utility of HMR, arguing that decreased viscosity may prolong the wait time for free energy crossing.^25^

An alternative to the HMR approach, which also exploits mass changes, aims at reducing the viscosity of water and increasing its diffusion by making water molecules lighter. Walser et al. demonstrated that water viscosity can be in fact changed in the MD simulations by rescaling water mass.^26^ Later, Lin and Tuckerman showed that, if solvent and peptide side chain masses are reduced 10-fold, peptide transitions to the folded state are facilitated.^27^ A more modest, 50% uniform reduction of atom masses has been employed by Hansmann and co-workers to study peptide aggregation.^28^ Their findings revealed a significant, in some cases, up to an order of magnitude boost in conformational transitions. It is of note that a straightforward change in all-atom masses rescales system time and is therefore useful as long as it does not entail a rescaling integration step. Recently, Rosas Jimenez et al. designed a modified TIP3P model, TIP3P-F, which is 16 times lighter than “normal” water.^29^ They found that the new water model preserves structural and energetic properties of the solvated peptide and ubiquitin protein as well as of the POPC lipid bilayer. Importantly, TIP3P-F accelerates the conformational sampling of peptide and protein approximately 2-fold, while the gain in sampling for the lipid bilayer is ∼25%. Consequently, this model may reduce the computational burden in the simulations of protein folding thermodynamics but cannot be used to study folding kinetics due to artificially reduced viscosity. Although TIP3P-F utilizes HMR in addition to the overall mass reduction, we refer to this model as light water (LW).

Despite significant progress in the methodology improving conformational sampling, converged MD simulations remain a serious challenge. In this article, we seek to address this challenge by examining two outstanding questions. First, previous studies have introduced HMR and LW in the context of constant temperature simulations under physiological conditions. However, the performance of these models in replica exchange simulations, commonly used in biomolecular modeling, is untested. Furthermore, HMR and LW may require revisions due to instabilities of integrator caused by the replica exchange method.^24^ Second, which of the two models, HMR or LW, offers the fastest rates of conformational sampling. To provide answers to these questions, we performed replica exchange with hybrid tempering (REHT) simulations, combining them with HMR or LW models. As a test system, we selected alanine dipeptide solvated in water. We determined the maximum integration steps, which ensure stability of REHT simulations with HMR and LW. To this end, we introduced scaling of integration steps with REHT temperatures. We then assessed the ability of REHT simulations with HMR and LW models to faithfully reproduce the structural and energetic properties of alanine dipeptide. Finally, we evaluated computational efforts needed to equilibrate the alanine dipeptide in HMR and LW models.

## 2 Models and Methods

### 2.1 Simulation System

This study demands a simple simulation system providing fast and accurate evaluation of different hydrogen mass repartitioning (HMR) and light water (LW) models. Consequently, we selected a single alanine dipeptide solvated in water (Figure 1). The system contained 809 water molecules and had a cubic shape with an edge of approximately 29 Å. The peptide N- and C-termini were capped with methyl groups, setting the charge of alanine dipeptide to zero. The all-atom CHARMM22 force field with CMAP corrections was applied to the peptide,^30^ whereas water was represented using the modified TIP3P model.^31,32^

**Figure 1:**
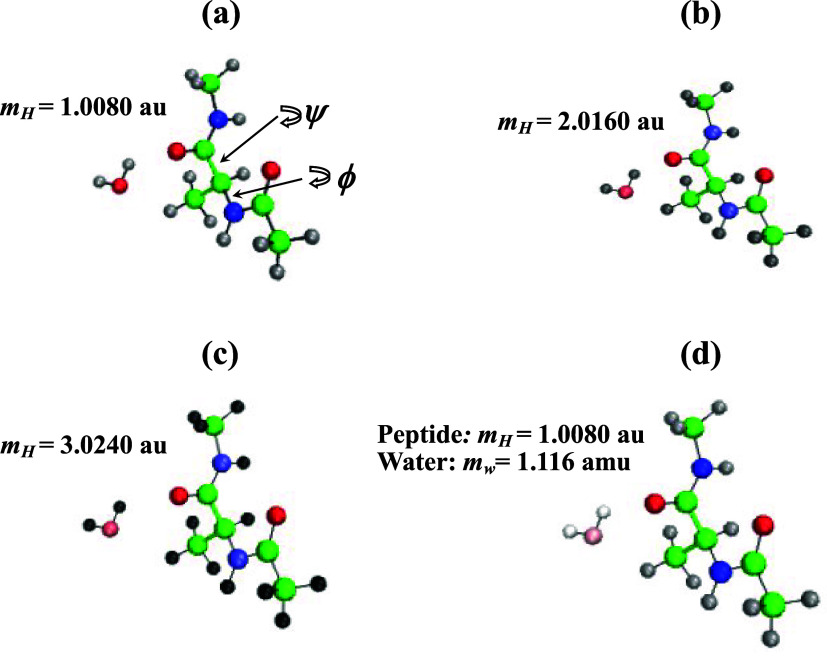
Snapshots of alanine dipeptide and water illustrate changes in the atomic masses in the four models: HMR1 (a), HMR2 (b), HMR3 (c), and LW (d). The shade of gray of hydrogen atoms depends on their mass *m_H_*: light gray in HMR1 with standard mass, gray in HMR2 with doubled mass, and dark gray in HMR3 with tripled mass. Pale color of the LW water molecule reflects a 16-fold reduction in its mass compared to standard water. For simplicity, this representation ignores repartitioning of hydrogen mass in LW and in heavy atoms. The locations of dihedral angles ϕ and ψ are shown in panel (a).

### 2.2 Selecting the Masses of Hydrogen Atoms and Water Molecules

We applied HMR to the simulations of alanine dipeptides in water. As a reference, we always considered the model with standard hydrogen mass *m_H_* = 1.0080 amu termed HMR1. The second HMR model referred to as HMR2 has hydrogen mass doubled to *m_H_* = 2.0160 amu, while the third denoted as HMR3 triples it to *m_H_* = 3.0240 amu (Figure 1). To keep the overall mass of molecules unchanged, the masses of heavy atoms covalently bonded to hydrogens were reduced. Mass repartitioning was applied to all hydrogen atoms in the system, including water and the peptide. For example, in HMR3, the mass of water oxygen atom was set to *m_O_* = 9.952 amu. Following previous study of Hopkins et al., we did not consider further mass repartitioning because it would result in methyl carbons being lighter than hydrogens.^21^ In addition, we applied a recently developed light water (LW) model, TIP3P-F, to alanine dipeptide simulations.^29^ In this model, mass changes are restricted to water, making it 16 times lighter. Specifically, the water hydrogen mass was set to *m_H_* = 0.186496 amu, whereas the water oxygen mass was *m_O_* = 0.743963 amu. As a result, the mass of the water molecule *m_w_* was reduced to 1.116 amu compared to the original value of 18 amu. It is of note that the TIP3P-F model also utilizes mass repartitioning, setting the ratio of oxygen to hydrogen masses equal to that in HMR3.

Because our simulations are conducted using NAMD,^33^ care must be taken in the implementation of LW or HMR. In principle, it should entail simple changes in the atomic masses present in the protein structure file (psf). However, the current build of NAMD3.0.2 is limited in its ability to handle some mass changes. First, any atom with a mass <1 amu is treated as either a Drude particle or a lone pair prohibiting the use of LW. Second, any oxygen atom that does not have a mass between 14 and 18 amu is not identified for SETTLE restraints. To mitigate these concerns, we patched NAMD so the original psf without LW or HMR could be used to correctly assign atom types before the masses are updated consistent with a LW or HMR. Our patch is available at https://github.com/KlimovLab/NAMD_with_mass_updates and is required for the proper use of HMR or LW.

### 2.3 Conformational Sampling

To test the utility of HMR and LW models in replica exchange simulations, we applied the replica exchange with hybrid tempering (REHT).^34^ Because its details are well-documented in our previous studies,^35^ we only provide below a brief overview. REHT introduces *R* replicas of a molecular system each subject to a unique condition *m* (0 ≤ *m* ≤ *R* – 1). These conditions are represented in part by temperatures *T_m_*. REHT implements selective tempering of solute and solvent using the following enthalpy

1 $$H_{m}=E_{p}+\sqrt{\frac{\beta_{m}^{\prime }}{\beta_{m}}}E_{\mathrm{ps}}+\frac{\beta_{m}^{\prime }}{\beta_{m}}E_{s}+\mathrm{PV}$$

where *E*_p_, *E*_ps_, and *E*_s_ are the energies of solute, solute–solvent, and solvent interactions, β_*m*_ = 1/(*R*_*c*_ *T*_*m*_), and *R*_*c*_ is the gas constant. The parameter β_*m*_^′^ introduces the second set of *R* temperatures *T*_*m*_^′^ < *T*_*m*_ if *m* > 0 and *T*_0_^′^ = *T*_0_, which control solvent tempering. Then, according to eq 1, REHT fully tempers solute as standard replica exchange does but applies limited tempering to the solvent by scaling up its interactions. Consequently, the temperature of solute is *T*_*m*_, whereas the effective temperature of solvent is *T*_*m*_^′^ meaning that the solvent is “colder” than the solute at all conditions *m* > 0. Thereby, the REHT condition *m* is defined by a pair of temperatures (*T*_*m*_, *T*_*m*_^′^). Partial tempering of solvent reduces the number of conditions *R* needed to span a temperature range. In our REHT simulations, solute includes peptide, and solvent consists of water. Using the Metropolis criterion, replicas *r* and *r*+1 at adjacent temperatures *T*_*m*_ and *T*_*m*+1_ are exchanged with the probability ω = *min*(1, *e* ^–Δ^), where Δ = β_*m*_(*H*_*m*_(*x*_*r*+1_) – *H*_*m*_(*x*_*r*_)) + β_*m*+1_(*H*_*m*+1_(*x*_*r*_) – *H*_*m*+1_(*x*_*r*+1_)), *x*_*r*_ and *x*_*r*+1_ are the coordinates of replicas *r* and *r*+1. Replica swaps produce a random walk of replicas over conditions of *m*. With properly selected interval between exchange attempts, REHT maintains equilibrium in the system while generating “colder” distribution of solvent states.

To stabilize HMR or LW simulations at elevated REHT temperatures, we applied the integration step scaling. As long as the integration step *δt* is set to 1 fs, replica exchange simulations may apply it across all temperature conditions up to 440 K without stability concerns.^36^ However, it has been found that replica exchange simulations with HMR model and *δt* = 5 fs become unstable at high temperature conditions.^24^ With this finding in mind, we scaled *δt* at REHT conditions as follows. We required the average atomic displacements at the lowest and given REHT temperatures, *T*_0_ and *T*_*m*_, to be the same, i.e., *δt*(*T*_0_) *v*(*T*_0_) = *δt*(*T*_*m*_) *v*(*T*_*m*_), where *v*(*T*) is the average thermal velocity at temperature *T*. Using the equipartition theorem, we find that the integration step at the REHT condition *m* is $\delta t\left(T_{m}\right)=\left[v\left(T_{0}\right)/v\left(T_{m}\right)\right]\delta t\left(T_{0}\right)=\sqrt{T_{0}/T_{m}}\delta t\left(T_{0}\right)$ . The scaled integration steps at 430 K are given in Table 1.

**Table 1:** Integrator Failures for HMR Models

model	temperature, *K*	*δt*_f_, fs	*δt*_s_, fs
HMR1	310	4.8	1.0
HMR2	310	6.0	3.5
HMR3	310	>6.0	4.0
HMR1	430	4.2	1.0
HMR2	430	5.6	3.0^a^
HMR3	430	6.0	3.4^a^

a Scaled due to simulations at an elevated temperature.

REHT or constant temperature simulations for probing integration steps (see the next section) were performed used NAMD.^33^ In the case of REHT, we used in-house scripts to manage replica exchanges. REHT simulations of alanine dipeptide utilized *R* = 8 conditions (*T*_*m*_, *T*_*m*_^′^), in which the temperatures *T*_*m*_ were distributed exponentially in the range from *T*_0_ = 310 to *T*_*R*–1_ = 430 K. The effective temperatures of solvent *T*_*m*_^′^ were distributed from *T*_0_^′^ = 310 to *T*_*R*–1_^′^ = 350 K. The complete list of conditions (*T*_*m*_, *T*_*m*_^′^) is given in Table S2. Replica swaps were attempted every 2 ps at 310 K for all values of integration steps *δt*. Due to *δt* scaling, the intervals between exchange attempts at higher REHT temperatures were shorter. All simulations used isobaric–isothermal (NPT) ensembles. The pressure was kept at 1 atm by using the Nosé–Hoover Langevin piston method. The piston period and decay times were 400 and 200 fs, respectively. Underdamped Langevin thermostat with a damping coefficient γ = 5 ps^–1^ maintained the temperature. System dimensions were coupled, and periodic boundary conditions were applied. Electrostatic interactions were computed with Ewald summations. van der Waals interactions were switched off by using the force switching in the interval from 8 to 12 Å. In HMR and LW simulations, the Verlet lists were updated every 20th integration step, while long-range electrostatic interactions were evaluated every second (in HMR) or fourth (in LW) integration step. SHAKE/SETTLE were used to constrain the hydrogen-related covalent bonds in HMR1 and LW models. Due to NAMD limitations in the unpatched version, HMR2 or HMR3 models forced SHAKE application to all hydrogen-related bonds, including those in water.

To prepare initial conditions for alanine dipeptide REHT or constant temperature simulations, we heated the system to the respective temperature and equilibrated it using the NPT ensemble for 100 ns. In all, we produced 3 REHT trajectories each of lengths of 100 ns. Analysis of simulation convergence presented in the Supporting Information (SI) indicated a rapid equilibration for all HMR and LW models, permitting us to treat all collected data as equilibrated. Therefore, for each HMR or LW model, we collected 300 ns of sampling at 310 K. The implications of rapid equilibration of alanine dipeptide are discussed in the SI.

### 2.4 Selecting Integration Steps

Following the findings of Feenstra et al., we selected the stable integration steps *δt*_*s*_ by monitoring crashes in MD trajectories.^19^ Specifically, for HMR models, we produced 120 NPT trajectories of 1 ns. The temperature in these simulations was set constant to either 310 or 430 K, while *δt* was incremented from 2.0 to 6.0 fs with a step of 0.2 fs. The temperature of 430 K corresponded to the highest REHT temperature. For a given model and integration step, the simulation trajectories used different initial structures prepared as described in the previous section. However, to minimize variance due to initial conditions, the starting structures were reused across different HMR models and *δt*. For each model, we determined the smallest integration step *δt*_*f*_, which results in at least one integrator failure in the ensemble of trajectories.

### 2.5 Computation of Structural Probes

To verify that HMR and LW models maintain the structural properties of alanine dipeptide, we used a number of probes. A hydrogen bond between the donor D and acceptor A atoms occurs if the distance between them is less than 4 Å and the angle ∠DHA >150°. This definition was applied to detect hydrogen bonds between water and the peptide. To probe hydration of alanine dipeptide, we used the radial distribution functions (RDF) centered at backbone oxygen or amide hydrogen atoms tracking water oxygen atoms. These RDF *g*(*r*) report the local number density of water at distance *r* from the reference atom and were averaged over oxygen or hydrogen atoms present in the peptide backbone. To compute the number of water molecules ⟨*N*_*w*_⟩ in the first solvation shell around the peptide, we assumed that it is composed of water with oxygen atoms being within 3.75 Å of any peptide heavy atom.

The energetic properties of the alanine dipeptide were probed in two ways. First, we evaluated the distributions of the peptide potential energies. Because SETTLE incorporates an H–H bond into a water molecule to make it rigid, while SHAKE utilizes a bond angle potential, the potential energies of the HMR1, HMR2, HMR3, and LW systems cannot be directly compared. Therefore, we computed the energy of alanine dipeptide *E*_pep_ in water using NAMD functionality namd_energy during simulation postprocessing. The energy *E*_pep_ includes intrapeptide bonded and nonbonded terms as well as nonbonded interactions between the peptide and water. Second, we computed the potential of the mean force (PMF) for the peptide. To this end, we considered the dihedral angles ϕ and ψ defined in Figure 1a. The probabilities *P*(ϕ) and *P*(ψ) to sample the angles ϕ or ψ were computed by using the angle bins of 12°. Then, the PMF *G*(ϕ) = −*R*_*c*_ *T* ln (*P*(ϕ)), where *T* = 310 K. The PMF *G*(ψ) is defined in a similar way. The equilibrium states Φ1 or Φ2 occurring along the ϕ dihedral angle and Ψ1 or Ψ2 occurring along ψ are introduced in Results and Discussion.

To evaluate the rates of equilibrating the peptide conformational ensemble, we considered the time dependencies *P*(Φ1; *t*) and *P*(Ψ1; *t*), which track the probabilities of occupancy of states Φ1 and Ψ1 at REHT time *t* and 310 K. These dependencies were fit with biexponential functions *a*_0_–*a*_1_ exp­(−*t*/τ_1_)–*a*_2_ exp­(−*t*/τ_2_), where *a*_0_, *a*_1_, *a*_2_, τ_1_, and τ_2_ are fitting parameters. The equilibration time scale τ is defined as REHT time, at which *P*(Φ1; *t*) or *P*(Ψ1; *t*) fits reach 95% of the respective equilibrium probability *P*(Φ1) or *P*(Ψ1). The computational effort required to equilibrate the peptide conformational ensemble was assessed using the time *t*_σ_ = *t*/σ scaled by the integration step-up factor σ = *δt*_s_/1 fs, where *δt*_s_ is the integration step used in the HMR or LW models. The respective equilibration time scale is defined as τ_σ_ = τ/σ.

## 3 Results and Discussion

The objective of this study is to systematically test and compare the performance of the HMR and LW models in replica exchange simulations. We begin our investigation with the selection of integration steps.

### 3.1 Selecting the Integration Step for Hydrogen Mass Repartitioning

To benefit from the application of HMR, we need to determine the largest integration step, *δt*_s_, which still ensures the stability of REHT simulations. Using the approach outlined in Models and Methods, we determined the smallest integration step *δt*_f_, which results in at least one integrator failure in the ensemble of MD trajectories. The results for alanine dipeptide simulations are listed in Table 1.

Table 1 shows that *δt*_f_ are model-dependent. Indeed, for HMR1 and at 310 K, the first integrator instabilities in the alanine dipeptide system occur at *δt*_f_ = 4.8 fs. As expected, at 430 K, *δt*_f_ is reduced to 4.2 fs. This outcome is approximately consistent with the previous reports that the HMR1 model with LINCS restraints fails at the integration steps exceeding 3 fs.^19,21^ Consequently, as a conservative reference for comparing HMR and LW models, we set *δt*_s_ to 1.0 fs for HMR1. Importantly, there is no need to scale *δt*_s_ in REHT, when HMR1 is used. As one may expect from previous studies, HMR2 delays integrator instabilities at 310 K to 6.0 fs. At 430 K, *δt*_f_ is lower being 5.6 fs. According to Table 1, we select *δt*_s_ for HMR2 to be 3.5 fs at 310 K. Following Models and Methods, *δt*_s_ at 430 K must be scaled by the factor $\sqrt{T_{0}/T_{R-1}}=0.85$ resulting in 3.0 fs. This selection of *δt*_s_ for HMR2 ensures integrator stability at all REHT temperatures. Further repartitioning of hydrogen mass in the HMR3 model sets *δt*_f_ in excess of 6 fs at 310 K, whereas a higher temperature of 430 K reduces *δt*_f_ to 6.0 fs. Then, for HMR3, we chose *δt*_s_ to be 4.0 fs at 310 K, which is scaled down to 3.4 fs at 430 K. It follows from Table 1 that this choice of *δt*_s_ also preserves the integrator stability in the HMR3 model at all temperatures. To ensure accurate comparison between the models, the integration step *δt*_s_ for the LW model was set to 1 fs. Our preliminary tests have suggested that the integration time steps selected for REHT simulations of HMR2 and HMR3 models are transferable to other systems, such as those involving peptides binding to lipid bilayers. However, if REHT temperature distribution requires changes or the algorithm for constraining rigid bonds is altered, additional investigation of integrator stability similar to that above is warranted.

### 3.2 Comparing Energetics of Solvated Peptide

With the stable integration steps *δt*_s_ set for all HMR and LW models, we turn to their systematic assessment in REHT simulations. First, for each model, we evaluate the average instantaneous temperature *T*_sim_ computed using the equipartition theorem and the number of degrees of freedom. According to Table 2, the average instantaneous temperature *T*_sim_ in any model deviates from the target 310 K by no more than 1.3 K or 0.4%. At 430 K, the deviation is slightly higher but does not exceed 2.8 K or 0.6%. We then analyze Maxwell–Boltzmann distributions (MBD) *P*(*E*_*k*_), where *E*_*k*_ is the kinetic energy of a system. Figure 2 shows these distributions for HMR and LW models at 310 K using all atoms. It is seen that for all four models, their *P*(*E*_*k*_) deviates from the theoretical distribution, albeit to a different degree. According to Table 2, HMR1 and LW exhibit the largest deviations measured by RMSD, whereas for HMR2 and HMR3 the deviation is almost 50% smaller. Similar results are seen at 430 K in Table 2. Independent of the model, the distributions *P*(*E*_*k*_) in Figure 2 overshoot the theoretical one at small *E*_*k*_ but underestimate it in the tails. Figure 2 also shows MBDs computed using heavy atoms. It is seen that the deviations from the theoretical *P*(*E*_*k*_) are smaller compared with the case when all atoms are considered. Furthermore, HMR1 reveals the smallest deviation followed by HMR2, HMR3, and LW. Their comparison suggests that the smaller the mass of atom engaged by the bond constraint, the larger the deviation from the theoretical distribution. To determine the source of these deviations, we repeated REHT simulations for the HMR1 model treating all hydrogen-related covalent bonds as flexible, i.e., we turned off SHAKE or SETTLE constraints. The resulting *P*(*E*_*k*_) computed for all atoms is in perfect agreement with the theoretical MBD that implicates rigid bonds as the source of MBD deviations. It is worth noting that for all models and temperatures, *T*_sim_ in Table 2 is lower than the target temperature. This systematic difference is explained by Figure 2, where *P*(*E*_*k*_) tails are consistently smaller than the theoretical MBD. Since the use of rigid bonds entails the deviations in *P*(*E*_*k*_), it also causes lower than expected *T*_sim_.

**Figure 2:**
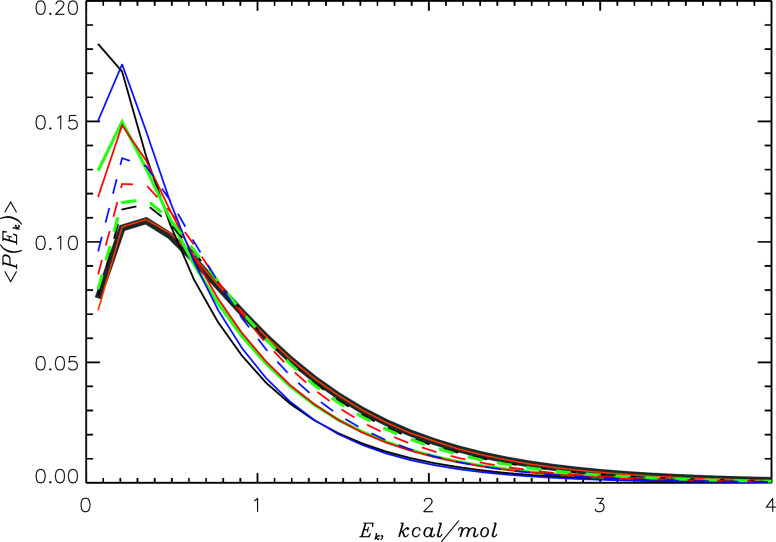
Maxwell–Boltzmann distributions *P*(*E*_*k*_) computed for all (solid lines) and heavy (dashed lines) atoms at 310 K REHT condition. Black, green, red, and blue colors represent the HMR1, HMR2, HMR3, and LW models, respectively. Theoretical *P*(*E*_*k*_) is shown by a gray thick solid line. The distribution computed for the HMR1 model without rigid bonds is given by a solid orange line. It shows an excellent agreement with the theoretical *P*(*E*_*k*_) indicating that rigid bond constraints cause deviations between theoretical and modeled *P*(*E*_*k*_). With hydrogens removed from computations, *P*(*E*_*k*_) approaches closer to the theoretical distribution.

**Table 2:** Comparing Temperatures and Kinetic Energies in HMR and LW Models

	310 K		430 K	
model	*T*_sim_, *K*	RMSD^a^, 10^–3^	*T*_sim_, *K*	RMSD^a^
HMR1	309.9 ± 0.4	1.4 ± 0.0	428.3 ± 0.4	1.9 ± 0.0
HMR2	308.7 ± 0.2	0.8 ± 0.0	427.2 ± 0.2	1.4 ± 0.0
HMR3	309.7 ± 0.3	0.7 ± 0.0	428.1 ± 0.4	1.4 ± 0.0
LW	309.5 ± 0.0	1.2 ± 0.0	429.3 ± 0.0	1.0 ± 0.0

a Root-mean-square deviation between the observed and theoretical Maxwell–Boltzmann distribution *P*(*E*_*k*_) for all atoms.

Next, we compare the distributions of potential energies *P*(*E*_pep_) of the alanine dipeptide in different models. As described in Models and Methods, the potential energy *E*_pep_ includes intrapeptide bonded and nonbonded terms *E*_pep,i_ and the energy of peptide–water interactions *E*_pep,w_. The distributions *P*(*E*_pep_) at 310 K are shown in Figure 3, whereas Table 3 lists their average values. The figure shows that all four models produce similar distributions *P*(*E*_pep_) at 310 K with very similar widths and locations of maxima. Indeed, we gather from Table 3 that the average peptide potential energies ⟨*E*_pep_⟩ in the HMR2, HMR3, and LW models are within the sampling error from the HMR1 reference. Decomposition of ⟨*E*_pep_⟩ reveals a more nuanced picture. The internal peptide energies ⟨*E*_pep,i_⟩ in HMR2 and LW models are within the error margin of the HMR1 reference, whereas HMR3 ⟨*E*_pep,i_⟩ is slightly higher. The average energy of peptide–water interactions ⟨*E*_pep,w_⟩ in the LW model differs from the HMR1 reference by a sampling error, but ⟨*E*_pep,w_⟩ for HMR2 and HMR3 models exhibits slightly lower values (by about 1%). It is of note that HMR2 and HMR3 ⟨*E*_pep,w_⟩ are lower than their HMR1 counterpart due to damping of fast water librational motions.^21^ However, this effect is refuted by straining of the peptide structure. In fact, the LW model has the least strained peptide conformation resulting in systematically lower energies *E*_pep_ seen in the distributions *P*(*E*_pep_) in Figure 3. Interestingly, the study of Hummer and co-workers also observed that the energy per molecule in the neat LW system is lower by about 0.4 kcal/mol than that of HMR1 reference.^29^

**Figure 3:**
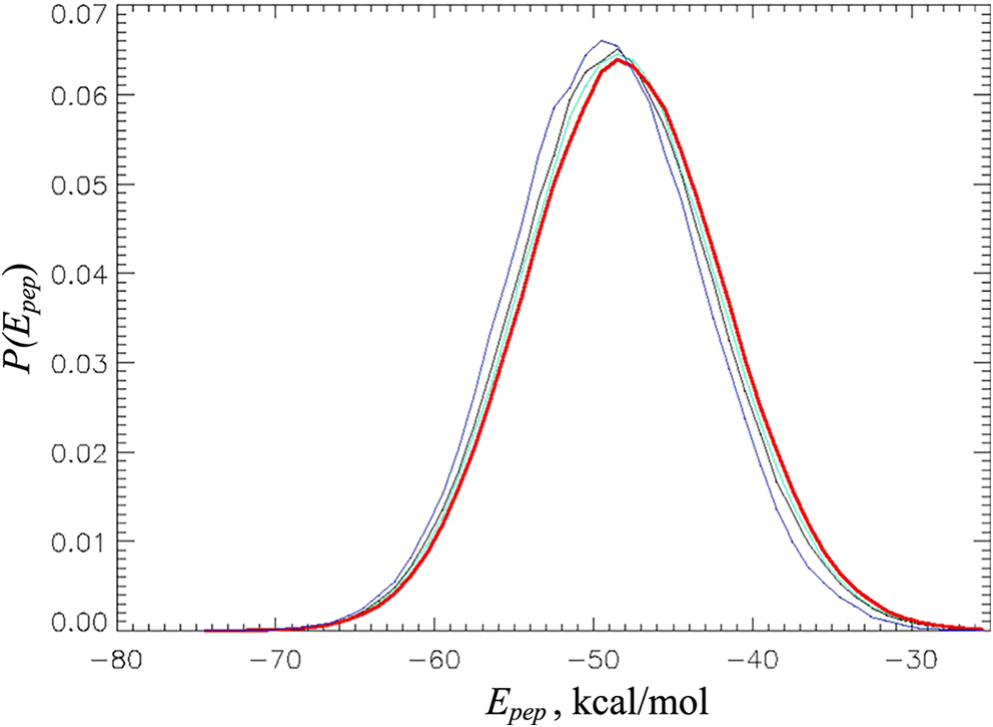
Distributions of potential energies *P*(*E*_pep_) of alanine dipeptide for HMR1 (black), HMR2 (green), HMR3 (red), and LW (in blue) models. This figure illustrates a good agreement between these distributions at 310 K.

**Table 3:** Peptide Potential Energies in HMR and LW Models

model	⟨*E*_pep,i_⟩, kcal/mol	⟨*E*_pep,w_⟩, kcal/mol	⟨*E*_pep_⟩, kcal/mol
HMR1	1.6 ± 0.6	–50.1 ± 0.1	–48.6 ± 0.6
HMR2	2.2 ± 0.2	–50.6 ± 0.0	–48.3 ± 0.2
HMR3	2.6 ± 0.2	–50.6 ± 0.0	–48.0 ± 0.2
LW	0.8 ± 0.0	–50.0 ± 0.0	–49.2 ± 0.0

The peptide free energy landscape is probed by computing the two PMF profiles, *G*(ϕ) and *G*(ψ), along the backbone dihedral angles ϕ and ψ (see Models and Methods). These profiles presented in Figure 4 show only minor variations. All PMF *G*(ϕ) are bimodal, revealing a global minimum at ϕ ≃ −66° and more shallow minimum at 54°. The plots for *G*(ψ) are also bimodal with minima at ψ ≃ 6 and 150°. To quantify the populations of these states, we assume that the peptide occupies a state, if its *G* < *G* ^†^ −0.5 kcal/mol, where *G* ^†^ is the maximum along the minimum free energy escape path from a state. With this definition, there are two states along the dihedral angle ϕ. State Φ1 corresponds to ϕ < −12 or 168° < ϕ, whereas state Φ2 resides within 24° < ϕ < 108°. Similarly, there are two states present along ψ, Ψ1 occurring within ψ < −144 or 96° < ψ, and Ψ2 with −60° < ψ < 48°. Table 4 contains the probabilities for alanine dipeptides to occur in these states. It follows from this table that the probabilities of all four states in HMR2 and HMR3 models agree within the margin of error with the HMR1 references. For LW and HMR1 models, the probabilities of Ψ1 and Ψ2 are close but differ beyond sampling errors, revealing 7 and 12% variance, respectively. Its origin is discussed below in the context of comparing hydrogen bonding between different models.

**Figure 4:**
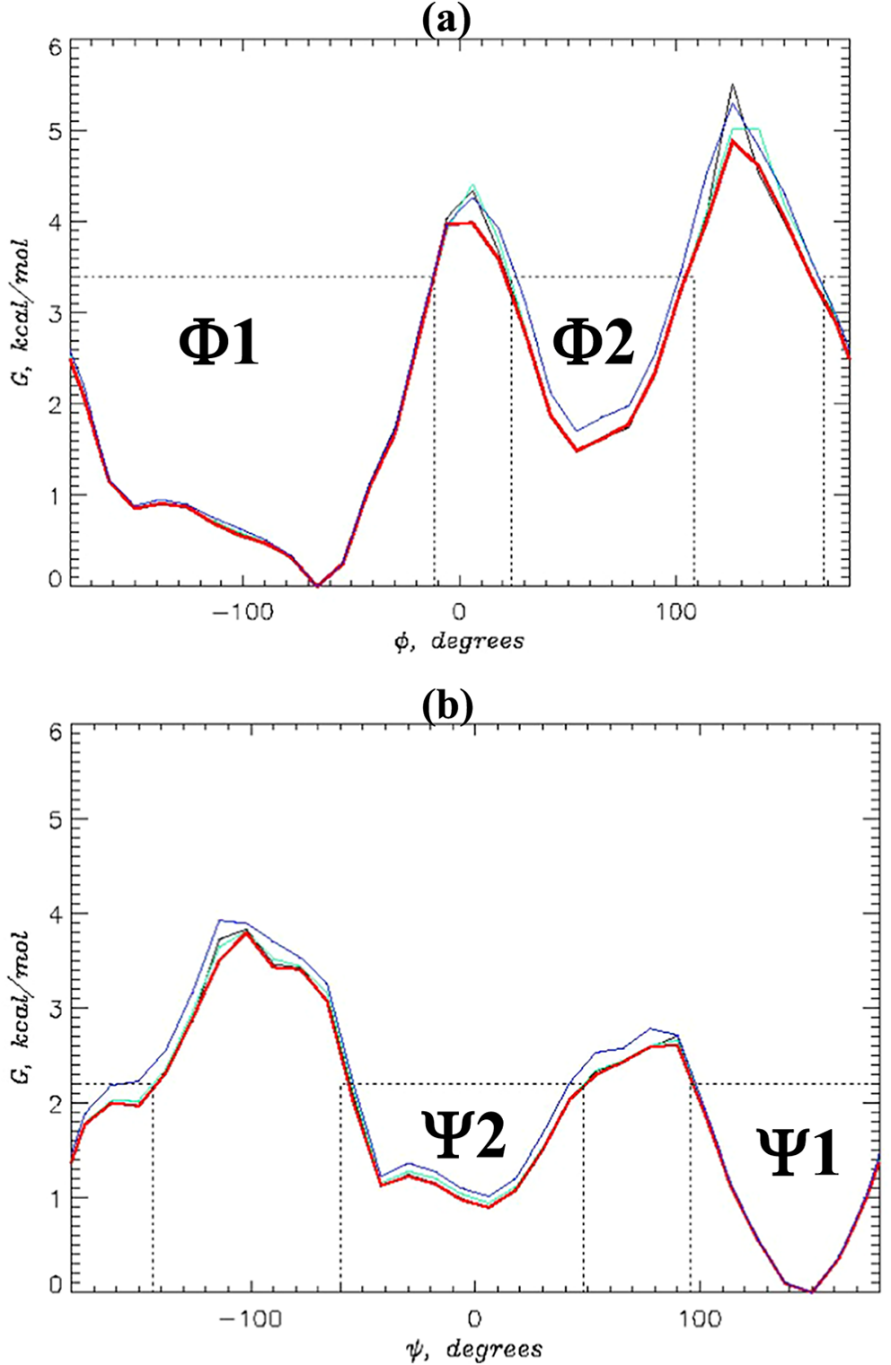
Potentials of mean force (PMF) *G*(ϕ) (a) and *G*(ψ) (b) computed along the peptide backbone dihedral angles ϕ and ψ at 310 K. Both panels demonstrate good agreement between PMF profiles computed for HMR1 (in black), HMR2 (in green), HMR3 (in red), and LW (in blue) models. The dotted lines mark the boundaries of the Φ1, Φ2, Ψ1, and Ψ2 states for HMR1.

**Table 4:** Probabilities of Dihedral Angle States in HMR and LW Models

model	*P*(Φ1)	*P*(Φ2)	*P*(Ψ1)	*P*(Ψ2)
HMR1	0.94 ± 0.01	0.06 ± 0.01	0.69 ± 0.01	0.26 ± 0.01
HMR2	0.93 ± 0.00	0.06 ± 0.00	0.70 ± 0.01	0.25 ± 0.00
HMR3	0.94 ± 0.01	0.06 ± 0.01	0.69 ± 0.00	0.26 ± 0.00
LW	0.95 ± 0.00	0.05 ± 0.00	0.75 ± 0.00	0.23 ± 0.00
hLW	0.94 ± 0.00	0.06 ± 0.00	0.69 ± 0.00	0.25 ± 0.00

### 3.3 Comparing Conformational Ensembles of Solvated Peptide

In addition to probing the energetic properties of the alanine dipeptide, we ascertain that HMR and LW models do not impact the peptide conformational ensemble. Following Models and Methods, we first compute the numbers of hydrogen bonds between the peptide and water. Table 5 lists their numbers formed by backbone carbonyl oxygens ⟨*N*_hb_(*O*)⟩ and nitrogens ⟨*N*_hb_(*N*)⟩. This table demonstrates a good agreement of HMR2 and HMR3 models with the reference HMR1. In fact, the number of hydrogen bonds does not deviate more than 4%. LW model exhibits somewhat lower counts of hydrogen bonds. Specifically, ⟨*N*_hb_(*O*)⟩ is reduced from the HMR1 reference by 17%, whereas the drop in ⟨*N*_hb_(*N*)⟩ is 8%. The likely source of these differences is the 3-fold higher diffusion coefficient reported for the LW model than for the standard TIP3P model used in HMR1.^29^ In addition, a lower count of hydrogen bonds formed by carbonyl oxygen compared to nitrogen may explain a larger deviation in LW probabilities of Ψ states from the HMR1 reference compared to Φ states. We also note that librational motions damped in HMR2 and HMR3 models compared to HMR1 have a negligible impact on peptide–water hydrogen bonding.

**Table 5:** Hydrogen Bonding and Hydration in HMR and LW Models

model	⟨*N*_hb_(*O*)⟩	⟨*N*_hb_(*N*)⟩	⟨*N*_w_⟩
HMR1	2.4 ± 0.0	1.2 ± 0.0	15.5 ± 0.0
HMR2	2.3 ± 0.0	1.2 ± 0.0	15.7 ± 0.0
HMR3	2.3 ± 0.0	1.2 ± 0.0	15.7 ± 0.0
LW	2.0 ± 0.0	1.1 ± 0.0	13.9 ± 0.0
hLW	2.4 ± 0.0	1.2 ± 0.0	15.4 ± 0.0

To investigate the density of water near peptide hydrogen bond donors and acceptors, we computed the radial distribution functions (RDF) as described in Models and Methods. Figure 5a shows the function *g*(*r*) probing the water number density around the peptide backbone oxygen atoms. The corresponding plots of *g*(*r*) reporting the water number density near the amide hydrogen are displayed in Figure 5b. Both panels demonstrate remarkable consistency between the four models, which includes the positions and amplitudes of water density maxima. Their values can be found in Table S2. To explore the overall hydration of alanine dipeptide, we computed the average numbers of water molecules ⟨*N*_w_⟩ in its first solvation shell for all four models as described in Models and Methods. The resulting data are summarized in Table 5. It is seen that ⟨*N*_w_⟩ for HMR models are nearly identical, but the LW value is about 10% lower than that for HMR1. Thus, these computations ascertain a near-perfect agreement in water density between HMR models, whereas LW consistently exhibits slightly weaker solvation.

**Figure 5:**
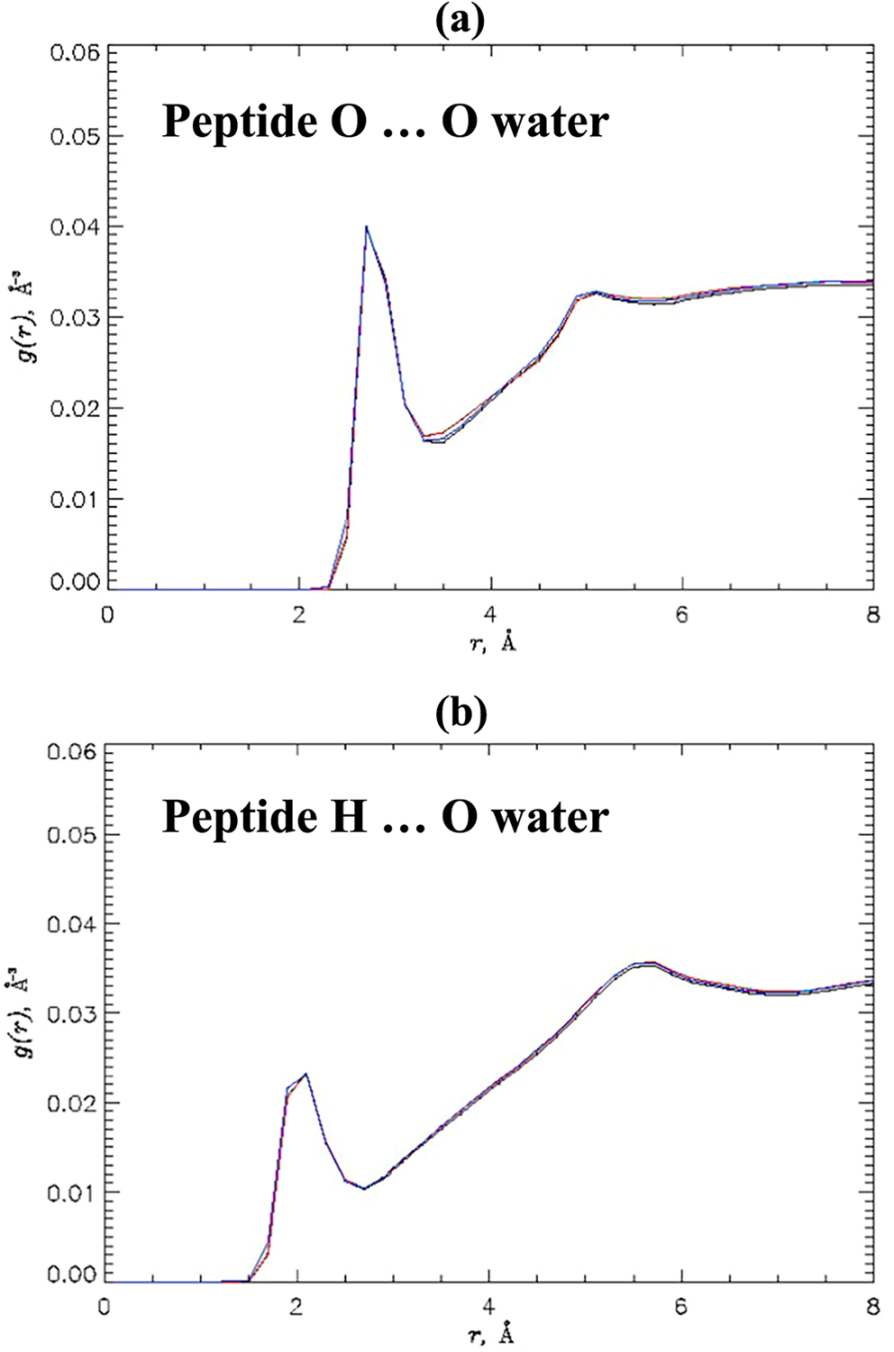
Radial distribution functions (RDF) *g*(*r*) probe water number density at the distance *r* from the peptide backbone oxygens (a) and amide hydrogens (b). The figure implicates a perfect consistency among *g*(*r*) for HMR1 (in black), HMR2 (in green), HMR3 (in red), and LW (in blue) models.

Finally, we directly compare the alanine dipeptide conformational ensembles by computing the probability distributions *P*(RMSD) of all-against-all root-mean-square deviations for the HMR and LW backbone conformations. The resulting plots are shown in Figure 6. The figure reveals excellent agreement among the four models, all displaying bimodal distributions. The two peaks in all four *P*(RMSD) are located at RMSD = 0.23 and 1.2 Å. Furthermore, the average RMSDs for HMR1, HMR2, HMR3, and LW models are 0.76, 0.76, 0.76, and 0.75 Å, respectively, again showing near-perfect agreement.

**Figure 6:**
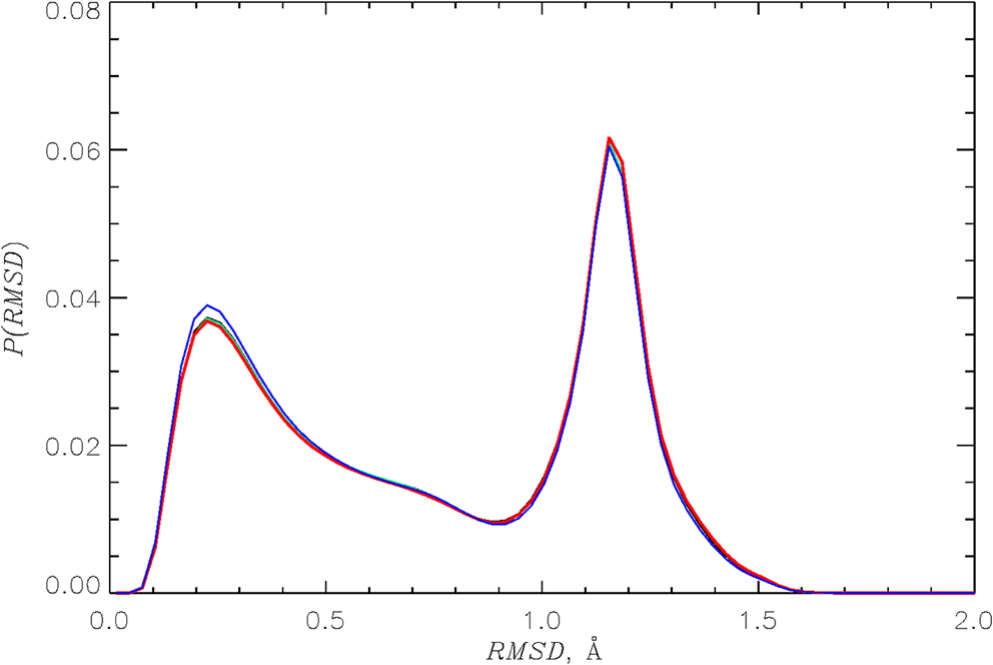
Probability distributions *P*(RMSD) of root-mean-square deviations for HMR1 (black), HMR2 (green), HMR3 (red), and LW (blue) backbone conformations. All-against-all RMSD structurally compare pairs of alanine dipeptides revealing excellent consistency of peptide conformational ensembles sampled by HMR and LW models.

### 3.4 Comparing Rates of Conformational Sampling

In the previous sections, we argued that HMR and LW models provide a generally consistent description of peptide properties. However, this analysis does not examine the computational efficiencies of these models. To evaluate them, we first determined the time scales of alanine dipeptide transitions between dihedral angle states underlying the equilibration process. Specifically, we followed REHT sampling at 310 K starting with the minor states Φ2 or Ψ2 and tracked the transitions to the dominant states Φ1 or Ψ1. We selected the transitions Φ2 → Φ1 and Ψ2 → Ψ1 to monitor equilibration because the states Φ1 and Ψ1 offer better numerical consistency across the models, thus affording a more accurate computation of equilibration time scales. Furthermore, selecting two reaction coordinates provides a more robust assessment of equilibration. As described in Models and Methods, the approach to the equilibrium state was monitored using *P*(Φ1; *t*) and *P*(Ψ1; *t*), the probabilities of occurrence of states Φ1 and Ψ1 at REHT time *t*. We then measured Φ2 → Φ1 and Ψ2 → Ψ1 transitions with the scaled REHT time *t*_σ_. This quantity defined in Models and Methods reflects the computational effort spent on equilibrating the alanine dipeptide conformational ensemble.

Figure 7a,b presents *P*(Φ1; *t*) and *P*(Ψ1; *t*) for HMR and LW models, whereas Figure 7c,d shows the respective *P*(Φ1; *t*_σ_) and *P*(Ψ1; *t*_σ_). The equilibration data for the HMR and LW models are collected in Table 6. It follows from this table and Figure 7a that the equilibration time scales τ for Φ1 state are consistent within 17% variance across HMR models as expected for the systems with minor differences in solvent viscosities.^19^ Predictably, the LW equilibration time scale τ is 45% shorter than for HMR models due to a 3-fold higher diffusion coefficient of TIP3P-F water compared to standard TIP3P. Very similar results are seen for the state Ψ1 in Table 6 and Figure 7b. Indeed, HMR2 or HMR3 time scales τ vary by up to 20% relative to HMR1, but the LW τ is about 30% faster. The most interesting data in Table 6 and Figure 7 pertain to the scaled REHT times *t*_σ_ measuring the computational effort. Equilibrium with Φ1 state in HMR2 and HMR3 models is established on the time scales τ_σ_ up to four times shorter than for HMR1. The LW model reveals τ_σ_ being only 1.8 times faster than that of HMR1. Nearly identical results follow from the analysis of the Ψ1 equilibration. To equilibrate this state, HMR2 and HMR3 models require up to about four times shorter τ_σ_ than HMR1, whereas the LW gain is 1.5-fold. These acceleration factors for the LW model are approximately consistent with those reported by Hummer and co-workers.^29^ Consequently, with the simulation settings described in Models and Methods, the LW model is more than 2-fold less computationally efficient than HMR3. This outcome means that increasing the integration step provides faster sampling than boosting water diffusion by making it lighter.

**Figure 7:**
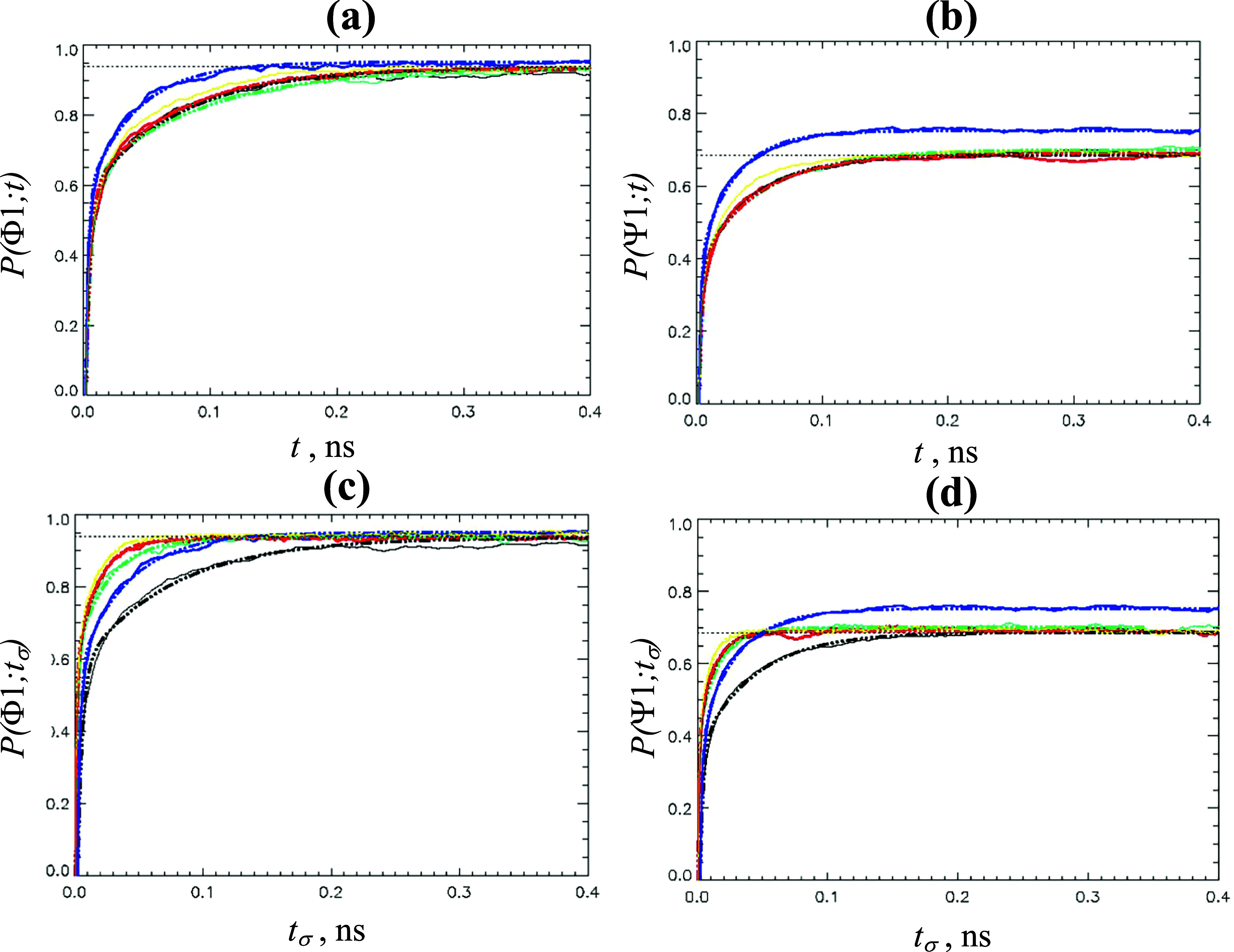
(a, b) Probabilities *P*(Φ1; *t*) (a) and *P*(Ψ1; *t*) (b) of occurrence of states Φ1 and Ψ1 at REHT time *t* and 310 K are shown for HMR1 (in black), HMR2 (in green), HMR3 (in red), LW (in blue), and hLW (in yellow) models. (c, d) Probabilities *P*(Φ1; *t*_σ_) (c) and *P*(Ψ1; *t*_σ_) (d) analogous to those in panels (a, b) are computed as a function of scaled REHT time *t*_σ_. The probabilities and their fits are shown by continuous and dot–dash lines. The horizontal dotted lines mark 95% levels of equilibrium probabilities of states Φ1 or Ψ1. The figure highlights the computational efficiencies of the HMR3 and hLW models.

**Table 6:** Equilibration Time Scales for HMR and LW Models

state	model	τ, ps	τ_σ_, ps	τ_σ,cor_ ^a^, ps
Φ1	HMR1	146.1	146.1	146.1
	HMR2	171.2	48.9	
	HMR3	136.4	34.1	40.9
	LW	80.9	80.9	45.7
	hLW	120.4	30.1	36.1
Ψ1	HMR1	94.3	94.3	94.3
	HMR2	113.1	32.3	
	HMR3	101.2	25.3	30.4
	LW	63.6	63.6	35.9
	hLW	94.0	23.5	28.2

a The time scales τ_σ,cor_ reflect the adjustments in computing LR interactions to provide their consistent evaluation across different integration steps.

It is important to note that many factors affect the comparison of computational efficiencies, including the frequency of computing long-range (LR) interactions. Following previous studies,^19^ we did not adjust their computation depending on the integration step keeping the settings applied in HMR1 (see Models and Methods). Nevertheless, to maintain consistency in their computation in HMR3 simulations against the HMR1 reference, we performed test simulations updating the Verlet list every fifth integration step and computing LR electrostatics every integration step. Our results showed that on average, these updates slow REHT execution by a factor of 1.2. The last column in Table 6 presents the time scales τ_σ,cor_ corrected by this adjustment. It follows then that compared to HMR1, HMR3 accelerates sampling to a slightly lesser degree, from 3.1 to 3.6 times. The LW model may utilize a 2 fs integration step, which also requires adjustments in computing LR interactions. To test LW performance with *δt*_s_ = 2 fs, we set the computations of Verlet lists to every 10th integration step and LR electrostatics to every second step. The REHT execution slowed down by 13% indicating that the time scales τ_σ,cor_ of LW conformational sampling with *δt*_s_ = 2 fs in Table 6 remain up to 18% slower compared to those of HMR3. It has to be mentioned that the acceleration of HMR3 and LW simulations relative to HMR1 is further affected by the REHT implementation. Utilizing native NAMD functionality for REHT would increase the time scales τ_σ,cor_ for HMR3 and LW and therefore reduce their gain to about 3-fold relative to HMR1. Taken together, these results and estimates argue that the outcome of the “competition” between the HMR and LW models does not qualitatively depend on the settings applied to computing LR interactions. Even if LR interaction computations are adjusted with the length of the integration step, the costs of their more frequent evaluations do not alter the performance ranking of HMR3 and LW.

Is it feasible to improve the computational efficiency of the LW model? This model already incorporates hydrogen mass repartitioning applied to water equivalent to that used in HMR3. If so, then one could attempt to increase the integration step *δt*_*s*_ in the LW simulations beyond 1 or 2 fs. However, with *δt*_s_ = 4 fs utilized by HMR3 at 310 K, this model causes integrator failure within 2 ps. Therefore, to bring it on par with HMR3 and sustain crash-free performance, one needs to increase the overall mass of water molecule by a factor of 4 and include the peptide in HMR. This hybrid model suggested by Hummer and co-workers^29^ is referred by us as hLW. Its REHT performance is probed in the SI. We compared hLW to other models by evaluating its free energy landscape, hydration, and computational efficiency. Table 4 shows that the equilibrium probabilities of all four dihedral angle states are in perfect agreement with the HMR1 reference. The same conclusion follows from a comparison of the numbers of hydrogen bonds and water molecules in the peptide first solvation shell. In fact, these numbers in Table 5 are almost indistinguishable between hLW and HMR1. Thus, hLW appears to correct for a slight drift of equilibrium probabilities observed for LW. Table 6 reveals that the equilibration time scale τ for the hLW model is up to 20% faster than of HMR1, but noticeably slower than that of LW reflecting increased water viscosity. More importantly, Figure 7 and Table 6 assess the computational efficiency of hLW. Independent of LR interaction settings, the hLW scaled time scales τ_σ_ or τ_σ,cor_ are somewhat faster than of HMR3. As expected with respect to τ_σ_, hLW is far more efficient than LW. Furthermore, even with the computation of LR interactions adjusted and the integration step set to 2 fs, LW remains notably less efficient than hLW. This assertion follows from comparing respective τ_σ,cor_ values in Table 6. Our analysis then points to three conclusions. First, an increase in LW water mass allowing us to increase the integration step is computationally advantageous. Second, hLW generally provides more accurate sampling of structural and energetic properties than LW. Third, because hLW and HMR3 differ only with respect to water mass, it appears that lighter water only marginally accelerates sampling compared to HMR3. Thus, taking into account sampling fidelity and computational efficiency of all models combined, we conclude that HMR3 is preferable because it (i) offers fast conformational sampling, (ii) accurately reproduces conformational ensemble and free energy landscape, and (iii) is more broadly applicable than LW variants. hLW is an excellent alternative to HMR3, but it is limited to “water-rich” systems. In fact, it has been shown that LW provides minor sampling improvements in the systems containing lipid bilayers.^29^

It is interesting to comment on the consequences of combining replica exchange with HMR. Concerns over integrator stability necessitate scaling down the integration steps at elevated REHT temperatures. Specifically, at *T*_*R*–1_ = 430 K, *δt*_s_ is reduced by 15% compared to 310 K. However, the coefficient of TIP3P water self-diffusion is reduced almost 2-fold even in a much more narrow temperature range from 310 to 350 K.^37^ This effect should compensate for the decrease in sampling rate due to scaled down *δt*_s_. Furthermore, enhanced water diffusion at high end REHT temperatures should enrich sampling at 310 K and thereby alleviate the decrease in water librational diffusion caused by heavier hydrogen atoms in HMR.

## 4 Conclusions

In this paper, we examined the utility and efficiency of hydrogen mass repartitioning (HMR) and light water (LW) models in the context of replica exchange with hybrid tempering simulations of alanine dipeptide. To maintain integrator stability, we introduced scaling of integration steps with REHT temperatures and determined their maximum lengths, assuring the stability of REHT simulations. We showed that HMR2 and HMR3 models featuring increased hydrogen mass and, to a lesser extent, the LW model reproduce the energetic and conformational properties of alanine dipeptide in water against the HMR1 reference. This conclusion is based on comparing kinetic and potential energies as well as the free energy landscapes of the peptide. The comparison also involved peptide structural properties, such as hydrogen bonding, water density in the peptide first solvation shell, and RMSD distributions. Thereby, our results demonstrate that both HMR and LW models can be integrated into replica exchange simulations. We then compared HMR and LW models with respect to computational efforts required to equilibrate alanine dipeptide. As expected, HMR2 and HMR3 are up to 4-fold more computationally efficient than the HMR1 reference, whereas LW lags behind being less than a factor two more efficient. As a result, LW efficiency is 2-fold lower than that of HMR3. This outcome means that increasing the integration step delivers faster sampling in comparison to boosting water diffusion. It holds true, albeit to a lesser extent, when the computation of long-range interactions is adjusted with the integration step, and its length is increased for the LW model. We considered a hybrid variant of LW, hLW, featuring heavier water and mass repartitioning applied to all hydrogens affording longer integration steps than LW does. hLW markedly improves computational efficiency and generally provides a more accurate reproduction of energetic and conformational properties of alanine dipeptide than LW. We concluded that although hLW slightly excels HMR3 in performance in replica exchange simulations, the latter is preferable due to its broad and more straightforward applicability. hLW remains an excellent alternative to HMR3, but is limited to “water-rich” systems. More generally, our findings suggest that among two approaches, HMR or decreasing water mass, the former is more effective.

## Supplementary Material



## Data Availability

NAMD is available at https://www.ks.uiuc.edu/Research/namd/. VMD is available at https://www.ks.uiuc.edu/Research/vmd/. Initial structures, topology files, NAMD configuration files, and codes used for data analysis are available at https://github.com/srbowers5/PGLA_MEM.

## Notes

Additional details concerning the model, methods, and results (PDF)
