# Supplementary material for: Accelerating Replica Exchange Molecular Dynamics: A Comparison of Hydrogen Mass Repartitioning and Light Water Models
Source: J Chem Theory Comput. 2026 Jan 9;22(2):1187–97. doi: 10.1021/acs.jctc.5c01929 (PMC12854735; doi:10.1021/acs.jctc.5c01929)
Supplement: Supplementary file 1 [file ct5c01929_si_001.pdf]

Supporting Information

Accelerating replica exchange molecular dynamics: A comparison of hydrogen mass repartitioning and light water models

Steven R. Bowers, William Jeffries, Christopher Lockhart, and Dmitri K. Klimov
School of Systems Biology, George Mason University, Manassas, VA 20110
E-mail: dklimov@gmu.edu

REHT setup: To evaluate the performance of hydrogen mass repartitioning and light water models, we used replica exchange with hybrid tempering (REHT) molecular dynamics. Description of REHT simulations is given in Models and Methods in the main text. The simulations used R=8 conditions (T_m,T'_m), where T_m is the temperature, T'_m is an effective temperature of solvent, and m is the condition index. The list of REHT conditions is given in Table S1.

Table S1: List of REHT conditions used in the simulations of alanine dipeptide.

m	T_m	T'_m
**0**	310.00	310.00
**1**	325.00	315.00
**2**	340.00	321.00
**3**	357.00	327.00
**4**	374.00	332.00
**5**	392.00	338.00
**6**	410.00	344.00
**7**	430.00	350.00

REHT performance: We assessed REHT technical performance adopting the approach from our previous studies [1]. REHT formalism requires random walks of replicas across the conditions. Fig. S1 shows that REHT simulations of alanine dipeptide in water indeed produced largely random walk of replicas across the conditions. Importantly, the figure does not reveal persistent trapping of replicas at any condition.

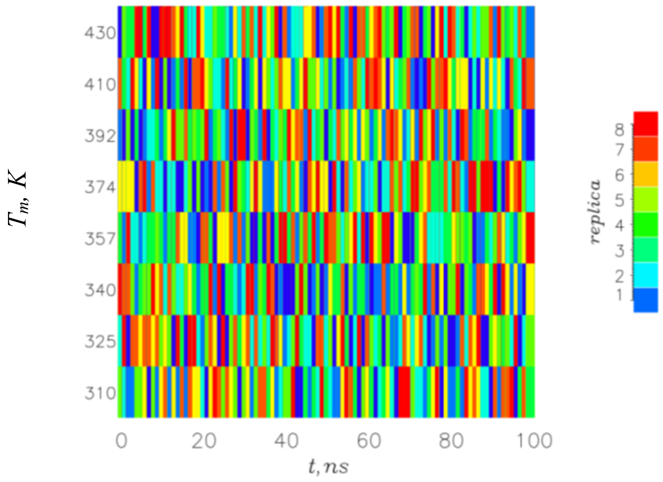

Figure S1 Traveling of replicas across the conditions m denoted by the temperatures T_m in the representative REHT trajectory. Colors are assigned to replicas at the start of the trajectory. Similar plots are seen for all models.

To probe the distribution of replicas across the conditions quantitatively, we computed the replica mixing parameter [2,3],

$$h(m) = 1 - \frac{\sqrt{\sum_{r=0}^{R-1} t_r^2}}{\sum_{r=0}^{R-1} t_r}, \tag{S1}$$

where m is the REHT condition and t_r is the time spent by replica r at m. If REHT produces a truly random distribution of replicas over all R conditions, $h(m)$ assumes the constant maximum theoretical value $h_o = 1 - 1/R^{1/2}$. For the alanine dipeptide simulations h_o is 0.64. Fig. S2 shows that in the REHT simulations $h(m)$ matches h_o perfectly. In fact, the average h across all m conditions is 0.64 for all models. Next, Fig. S3 presents the replica exchange rates $\alpha(m)$. For HMR1, HMR2, HMR3, and hLW the average $\alpha(m)$ is 0.49, but is reduced to 0.37 for LW. These rates are approximately in optimal range [4]. Taken together, Figs. S1-S3 suggest a good mixing of replicas across the conditions as expected from REHT for all models.

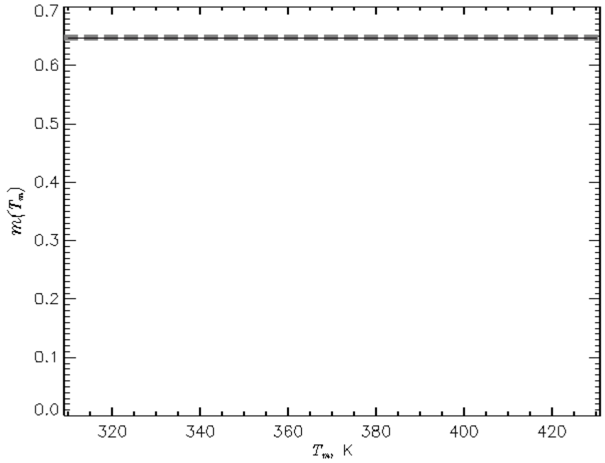

Figure S2 The replica mixing parameter $h(m)$ as a function of REHT condition m represented by temperature T_m. The maximum theoretical value h_o is marked by a dashed grey line, while HMR1 data are presented by black solid line. HMR2, HMR3, LW, and hLW data are omitted for clarity, because they are indistinguishable from the HMR1 reference. The data is averaged across REHT trajectories.

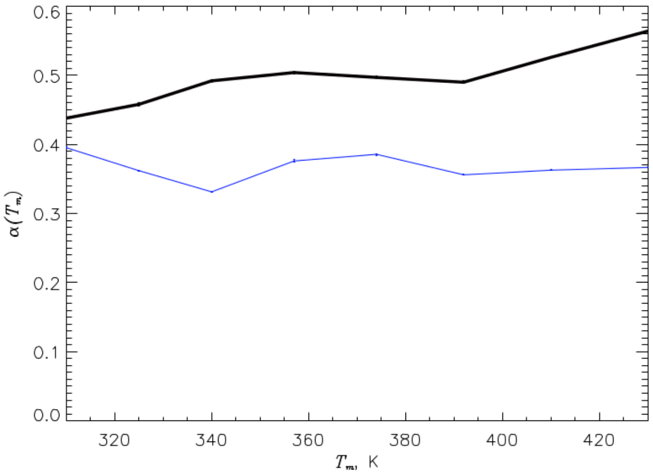

Figure S3 Replica exchange rates $\alpha(m)$ as a function of REHT condition m represented by temperature T_m. The HMR1 and LW data are shown in black and blue, respectively. The rates for HMR2, HMR3, and hLW are indistinguishable from the HMR1 reference. The rates $\alpha(m)$ are averaged across REHT trajectories.

Convergence of REHT sampling: To evaluate the convergence of REHT simulations, we monitored the probabilities $P(\Phi1;t)$ and $P(\Psi1;t)$ of occurrence of dihedral angle states Φ1 and Ψ1 at REHT time *t* and 310 K. Fig. S4 demonstrates that these probabilities for all models fluctuate around the respective baselines suggesting absence of equilibration process. Consequently, we treated all sampling collected, namely 300 ns per model, as equilibrated.

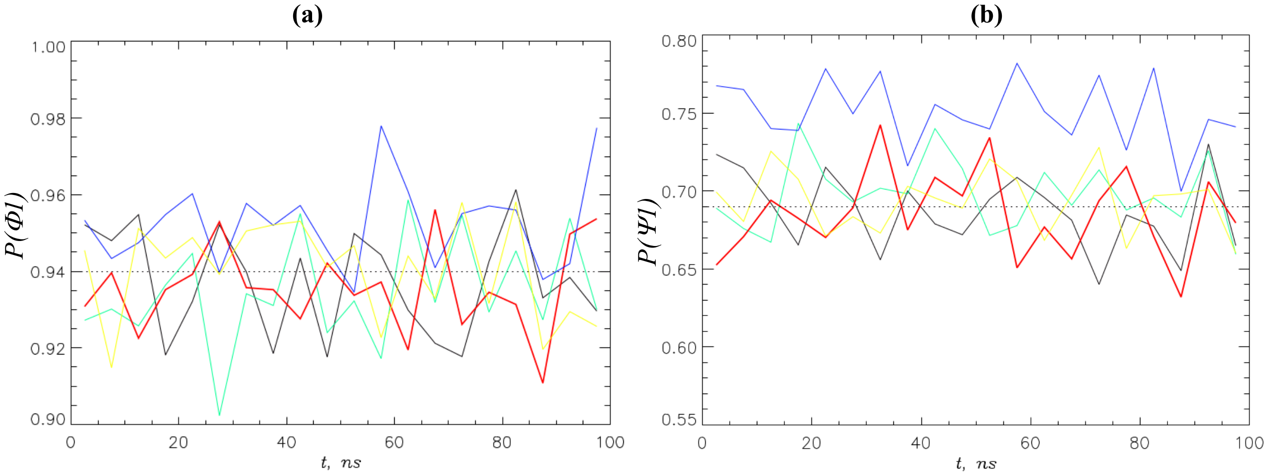

Figure S4 The probabilities $P(\Phi1;t)$ (a) and $P(\Psi1;t)$ (b) of occurrence of states Φ1 and Ψ1 at REHT time *t* and 310 K in alanine dipeptide simulations. The data for HMR1, HMR2, HMR3, LW, and hLW models are given in black, green, red, blue, and yellow. The figure reveals no discernible equilibration period in the REHT simulations.

It is important to discuss the implications of REHT convergence. Fast equilibration of alanine dipeptide, which contains relatively few atoms, implies that longer (> 1 μs) simulations would only reduce already small sampling errors in Tables 2-5 and minimize further the deviations between the plots in Figs. 4-6. However, none of the conclusions concerning the validity of HMR and LW models or their computational efficiency will be affected by longer sampling. For more complex systems, including peptides binding to a lipid bilayer, longer (> 1 μs) simulations are critical for robust conclusions.

Analysis of water radial distribution functions: To provide a quantitative assessment of water distribution near peptide donors and acceptors of hydrogen bonds, we analyzed the radial distribution functions $g(r)$ shown in Fig. 5. Specifically, these functions report the water number density near amide hydrogens and carbonyl oxygens in alanine dipeptide backbone. Table S2 lists the locations and amplitudes of the maxima in $g(r)$.

Table S2 Analysis of radial distribution functions probing water number density.

**Backbone atom**		**HMR1**	**HMR2**	**HMR3**	**LW**
**H**	r_{max}, Å[a]	2.1	2.1	2.1	2.1
	g_{max}, Å^{-3} [b]	0.023	0.023	0.023	0.023
**O**	r_{max}, Å[a]	1.9	1.9	1.9	1.9
	g_{max}, Å^{-3} [b]	0.074	0.072	0.073	0.072

[a] r_{max} is the location of water number density peak in $g(r)$.

[b] g_{max} is the value of water number density peak.

References:

[1] Bowers, S. R., Lockhart, C., and Klimov, D. K. (2023) Replica Exchange with Hybrid Tempering Efficiently Samples PGLa Peptide Binding to Anionic Bilayer. *J. Chem. Theor. Comput.* **19**, 6532–6550.

[2] Han, M. and Hansmann, U. H. E. (2011) Replica exchange molecular dynamics of the thermodynamics of fibril growth of Alzheimer's Aβ42 peptide. *J. Chem. Phys.* **135**, 065101.

[3] Nadler, W. and Hansmann, U. H. E. (2008) Optimized Explicit-Solvent Replica Exchange Molecular Dynamics from Scratch. *J. Phys. Chem. B* **112**, 10386–10387.

[4] Denschlag, R., Lingenheil, M., and Tavan, P. (2009) Optimal temperature ladders in replica exchange simulations. *Chem. Phys. Lett.* **473**, 193-195.
